# Pre-Operative Functional Mapping in Patients with Brain Tumors by fMRI and MEG: Advantages and Disadvantages in the Use of One Technique over the Other

**DOI:** 10.3390/life13030609

**Published:** 2023-02-22

**Authors:** Elisa Cargnelutti, Barbara Tomasino

**Affiliations:** Scientific Institute, IRCCS E. Medea, Dipartimento/Unità Operativa Pasian di Prato, 33037 Udine, Italy

**Keywords:** fMRI, MEG, pre-operative mapping, brain tumor, multimodal functional assessment

## Abstract

**Simple Summary:**

Providing an accurate localization of the brain functions before proceeding with neurosurgery is of vital importance to spare the removal of crucial brain areas. In this review paper, we investigated how two techniques, namely functional magnetic resonance imaging (fMRI) and magnetoencephalography (MEG), can serve this purpose, with specific reference to pre-operative assessment of sensorimotor and language functions in patients with brain tumors. First, we explored the methodological strengths and issues of each technique from data acquisition, to data processing and result display, also providing a temporal overview of these technical aspects and potential strategies developed to overcome the main issues. Then, we identified 16 studies that made pre-operative assessments by both techniques and accurately scrutinized them. Overall, despite the potential limitations associated with one technique or the other, the majority of these studies underlined the reliable and complementary use of fMRI and MEG. For these reasons, with the aim of the most reliable pre-operative assessment as possible, we recommend the combined use of both techniques, favored by the recent technological advances making MEG use more feasible.

**Abstract:**

Pre-operative mapping of brain functions is crucial to plan neurosurgery and investigate potential plasticity processes. Due to its availability, functional magnetic resonance imaging (fMRI) is widely used for this purpose; on the other hand, the demanding cost and maintenance limit the use of magnetoencephalography (MEG), despite several studies reporting its accuracy in localizing brain functions of interest in patient populations. In this review paper, we discuss the strengths and weaknesses of both techniques, from a methodological perspective first; then, we scrutinized and commented on the findings from 16 studies, identified by a database search, that made pre-operative assessments using both techniques in patients with brain tumors. We commented on the results by accounting for study limitations associated with small sample sizes and variability in the used tasks. Overall, we found that, although some studies reported the superiority for MEG, the majority of them underlined the complementary use of these techniques and suggested assessment using both. Indeed, both fMRI and MEG present some disadvantages, although the development of novel devices and processing procedures has enabled ever more accurate assessments. In particular, the development of new, more feasible MEG devices will allow widespread availability of this technique and its routinely combined use with fMRI.

## 1. Introduction

The accurate identification of the brain areas subserving a given brain function is a crucial process in neurosurgery planning. In recent decades, studies have adopted different non-invasive techniques for this purpose to primarily identify potential lesion proximity to eloquent areas of the language and sensorimotor systems. Additionally, due to extensive availability of magnetic resonance imaging (MRI) instrumentation for diagnostic purposes, functional MRI (fMRI) probably represents the most widely used technique. Besides fMRI, electroencephalography (EEG) and magnetoencephalography (MEG) have been employed too, although traditionally deputed to epileptic activity monitoring [1,2].

The principle of fMRI resides in the blood flow change associated with the hemodynamic response in brain areas involved in a given brain function; this generates a detectable signal, which is measured in terms of a blood-oxygen-level dependent (BOLD) contrast. On the other hand, EEG and MEG measure the brain activity directly, by recording, respectively, the electrical and magnetic fields generated by thousands of neurons that activate in synchrony.

EEG and MEG provide similar but not completely equivalent information about brain processes [3] and each technique has its advantages over the other. EEG recordings are more feasible, whereas recording by MEG is tricky and probably represents its major issue. The MEG apparatus requires distinguishing the brain’s magnetic activity (in the order of 10^−15^ T) from external interferences, especially the much stronger Earth’s magnetic field (10^−5^ T). Despite the need for a shielded room, super-conducting quantum interference detectors (SQUIDs) are also required and need a continuous cooling process by liquid helium (−270 °C). These factors make traditional MEG systems very demanding, being very bulky (with a weight of several tons) and very expensive, not only for the initial set up, but also for their maintenance.

Nevertheless, overall MEG is considered superior to EEG, because it normally has higher spatial accuracy related to the fact that magnetic versus electric fields are less distorted by the skull, scalp, and cerebrospinal fluid and because it better detects activities located in the gyri [4]. Further, it requires fewer neurons to be activated in synchrony to detect brain activity and source modeling, required to localize the detected field sources, is generally less complex and more accurate (for a comparison between EEG and MEG, see [5,6]).

**Figure 1:**
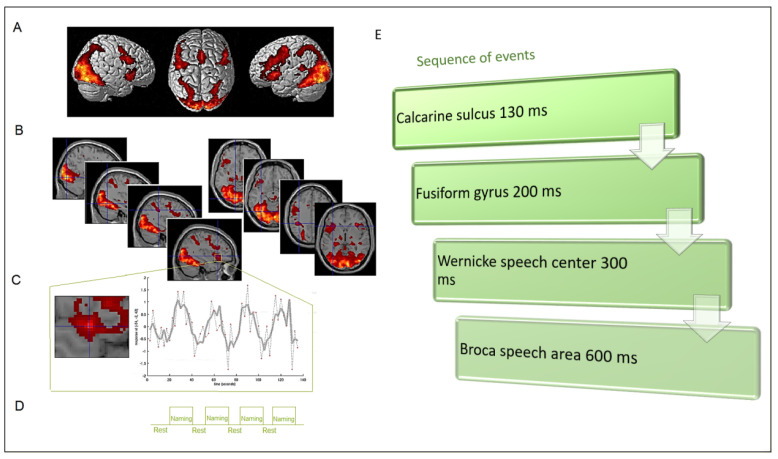
Example of spatial and temporal information provided by fMRI and MEG during a representative task (i.e., object naming) in pre-operative mapping. (**A**) fMRI functional overlay on a glass brain, providing the entire naming-related network (data from our lab, acquired by a 3 T tomograph). (**B**) Same fMRI activations overlaid on sagittal and axial structural images (note the relevant nodes represented by the calcarine cortex, fusiform gyrus, Wernicke’s, and Broca’s areas); these images can provide a hint on the distance between the activation center of mass and potential lesions. (**C**,**D**) Detail of one activation cluster (high-field scanners allow the acquisition of images at high spatial resolution) and related averaged fMRI signal, resulting from the overall activation across several task repetitions (i.e., blocks) throughout the whole task presentation. (**E**) Temporal fingerprints of the object-naming task, provided by MEG high temporal resolution (data from Grummich et al., 2006); temporal information can be relevant for planning the proper delay after stimulus presentation to be used during intra-operative stimulation mapping.

In light of these technical considerations, in the current review paper we aimed to compare fMRI and MEG in terms of brain activity localization, from the perspective of surgical planning for brain tumor resection (Figure 1). Indeed, as well as fMRI, when MEG data are merged with high-resolution MR images, the resultant magnetic source imaging (MSI) can provide accurate localization of the detected signal sources and enable functional intra-operative navigation to guide safe tumor resection. However, this combined approach is poorly used for several reasons, first the poor availability of the MEG apparatus in clinical services.

Several studies have independently addressed the accuracy of fMRI and MEG in the localization of cognitive and sensorimotor functions in patient populations, for instance comparing the results from those obtained by intra-operative mapping. Localizing the functional activations associated with a given function is crucial, because tumor development can cause brain remodeling through neuroplasticity processes and activations can be displaced with respect to the healthy brain (see [7,8]). Identifying the potentially new location of these activations is fundamental to spare the removal of essential brain areas during surgery.

Evidence of reliability in the use of fMRI or MEG alone has been widely reported. A recent meta-analysis [9] reported the accuracy of fMRI in language mapping by taking intra-operative recordings via direct cortical stimulation (DCS) as a reference. Here, the reported sensitivity ranged from 51 to 80% and specificity 25 to 82%. Bizzi and colleagues [10] suggested that accuracy can by influenced by glioma grade, as they observed a remarkably lower sensitivity in glioblastomas (i.e., high-grade tumors) than in low-grade gliomas (65 vs. 93%), although specificity was higher (93 vs. 79%). Concerning MEG, Ellis et al. [11] commented that studies did not typically report a quantitative assessment of accuracy in terms of sensitivity and specificity; nevertheless, this technique has been widely observed to provide a reliable assessment of pre-operative localization of brain functional activity [12,13,14,15].

In the current review, we will first provide an overview of the methodological strengths and weaknesses of each technique, to then compare them more directly by commenting the results reported in studies that adopted both of them for pre-operative brain activity assessment. The final aim was to shed light on the advantages and disadvantages in applying one methodology versus the other and the related recommendation about their use in clinical practice.

## 2. Methodological Comparison between fMRI and MEG

Overall, agreement exists in relation to the pros and cons associated with each technique, regardless of the specific findings observed in the pertaining studies. Apart from the factors representing limitations for the acquisition procedure, for instance the presence of metallic implants in patients (which may prevent fMRI acquisition and cause severe artifacts in the case of MEG), the two techniques present characteristics that influence the quality of the measured brain activity that can either represent a drawback or a strong point for that technique.

### 2.1. Approach to Measuring the Brain Activity

Executing a given task activates the related brain network, with all the recruited areas simultaneously displayed by fMRI. On the other hand, MEG records the temporal development of the whole brain process, hence its spatiotemporal fingerprints; it detects the earliest component of the evoked response arising from primary eloquent areas, which can be separated from later components mainly arising from non-primary areas [16]. This is generally considered as an advantage over fMRI, given that it enables distinguishing between primary activated areas and areas that secondarily support that function. Nevertheless, this is positive, as fMRI can more easily capture the whole network associated with a specific brain function and investigate how the brain has been reshaped to compensate for glioma growth.

### 2.2. Spatial and Temporal Resolution

Concerning resolution, the two techniques can be considered as complementary. The advantage of fMRI relies in the high spatial resolution of a few millimeters (depending on the voxel size), although a drawback is represented by its poor temporal resolution; indeed, BOLD responses are detected after several seconds following the activation of a given brain region and also lasts several seconds after deactivation.

On the other hand, traditional MEG is characterized by a higher temporal resolution (in the order of milliseconds) but a poorer spatial resolution, especially when the source of the detected signal is deep (for reviews, see [17,18,19]). Even when the source is highly localized, resultant images are typically blurred. Spatial resolution is not homogeneous across the brain and can be affected by several factors, including distance from the sensors and signal-to-noise ratio [20]. Poor spatial resolution is primarily determined by the fact that the number of sensors covering the brain is finite, therefore localizing the source that generated the detected signal is generally problematic (see afterwards). This is particularly characteristic of earlier MEG devices, only possessing a few channels covering a limited portion of the brain.

Furthermore, classical MEG systems could not detect subcortical activity; in fact, MEG mainly detects relatively superficial currents in the fissural cortex, being sensitive to sources tangential to the skull; nevertheless, more recent whole-head systems with hundreds of sensor can cover the whole head and can detect activity even in subcortical regions [21]. However, these systems still have limited coverage of prefrontal areas and the cerebellum, limiting the detection of brain activity in these areas [20].

In the case of presurgical mapping, having high spatial resolution is fundamental, making fMRI more appropriate for this purpose, at least in general terms. In the following paragraphs, we will compare and discuss the in-depth suitability of both techniques from the perspective of neurosurgery.

### 2.3. Issues with Signal Detection and Processing

One of the major drawbacks associated with fMRI is represented by the inflow effect. In reality, BOLD signal may be altered by the signal coming from the blood flow in the draining veins, for instance in those supplying the central sulcus; the result is an alteration in neurovascular coupling. This was especially observed when using the conventional 1.5 T system with a gradient-echo sequence [22,23]. Further, the signal can be affected in the presence of brain lesions such as arteriovenous malformations or brain tumors and associated edema. In these cases, lesion removal is likely to restore signal detection [23]. In the case of tumors, the effect is attributed to angiogenesis and altered permeability of the tumor vasculature, especially for high-grade lesions [24]. In the case of low-grade gliomas, the process can be attributed to angiogenesis as well, but also to cellular dysfunction associated with tissue infiltration [25,26].

Nevertheless, these issues are scaled down by scans performed at high magnetic-field strength (3 T or greater) and the use of a spin-echo or gradient-echo EPI sequence instead of the traditional gradient-echo sequence. These solutions, indeed, decrease the effect of the inflow effect and then increase the BOLD contrast associated with the measured brain process [22,23,27,28]. This suggests that signal quality is highly influenced by both acquisition devices and acquisition and processing procedures.

Acquisition devices also influence signal detection by MEG. Sensors for magnetic signal detection are typically represented by gradiometers (axial or planar), although these are rather insensitive to deep brain sources. The latter can be, instead, collected by magnetometers, but these are also more sensitive to ambient noise. Modern devices include both gradiometers and magnetometers and their combination significantly increases the signal-to-noise ratio (for reviews, see [19,21,29,30]).

For both fMRI and MEG, signal detection may be challenging in correspondence to specific brain structures. For instance, partial-volume effects in fMRI can hamper signal detection when activation is confined to one bank of the sulcus [30]. Conversely, MEG can fail in the detection of an extended signal, when it originates from opposite banks of a sulcus [31]. In such cases, using MEG and fMRI could, instead, navigate around the problem.

Concerning signal processing, the main issue concerns MEG and, specifically, the solution to the inverse problem for signal localization; in other words, after recording the magnetic current on the scalp, it is necessary to identify the correct location of the brain source that generated it. The inverse problem is defined to be ill-posed, as there is not a unique solution to it. The most commonly used source estimation approach is represented by the equivalent current dipole (ECD), which captures the activity of tens of thousands of cortical neurons that activate in synchrony. A single ECD source can represent a valid solution in the case of strong dipolar patterns with a high signal-to-noise ratio, as is the case of the early components of somatosensory-evoked fields [21], but is limited otherwise.

Alternative approaches have been developed to provide a more reliable resolution to the inverse problem; these include spatial filtering and appear even more useful when analyzing widespread activation patterns such as those related to language functions [32]. Some authors, however, have underlined that defining the actual extent of a source can still remain problematic [18]. A widely used spatial-filtering approach is represented by beamforming [33,34], which excludes signals external to the source of interest (however, with difficulty in the case of highly synchronous sources). Source estimation can also be performed by distributed inverse methods, including minimum-norm estimation (MNE, [35]), dynamical statistical parametric mapping (dSPM, [36]), and standardized low-resolution brain electromagnetic tomography (sLORETA, [37]).

Nevertheless, even signal detection by fMRI has to be evaluated, and this can vary depending on the software used for processing and the selected options. For instance, different basis functions can be used to model the hemodynamic response (e.g., hemodynamic response function, Fourier set, finite impulse response), potentially giving different results.

Finally, another relevant issue pertains to the spatial co-registration procedure to the anatomical MR image. This is likely to be more challenging and less accurate for MEG than fMRI [38], even though appropriate procedures are recommended for fMRI, in order to avoid misalignment [39,40]. Indeed, whereas the problem with the latter may be represented by potential distortion due to susceptibility effects, co-registration with MEG is highly prone to human error. Reported co-registration errors for MEG are typically about 5 to 10 mm [41,42], although recently developed technological advances and algorithms can decrease errors to a few millimeters [43]. The most traditional approach was initially represented by the registration to reference points represented by anatomical landmarks (i.e., nasion, inion, and bilateral pre-auricular points). However, by using specific algorithms, such as contour fitting, accuracy can be increased [38,44].

All these concerns have to be weighted in light of clinical requirements and timing. Indeed, although very promising, these approaches are still time-consuming and it is sometimes difficult to integrate them into the clinical routine, considering also that patients can become tired more easily following intensive pre-operative assessments. For this reason, the best practical solution should be, generally, the one that provides the most reliable assessment within a reasonable time.

## 3. Direct Comparison between fMRI and MEG in Pre-Operative Mapping

Following this methodological comparison between the two approaches, we now comment on the findings from studies assessing pre-operative functional activation in patients with brain tumors by means of both fMRI and MEG. In order to identify peer-reviewed papers on this topic, we ran a paper search on the PubMed, Web of Science, and Scholar databases using the following keywords: (fMRI OR “functional MR”) AND (MEG OR magnetoencephalography OR MSI OR “magnetic source”) AND (glioma OR tumor OR lesion) AND (surgery OR preoperative).

**Figure 2:**
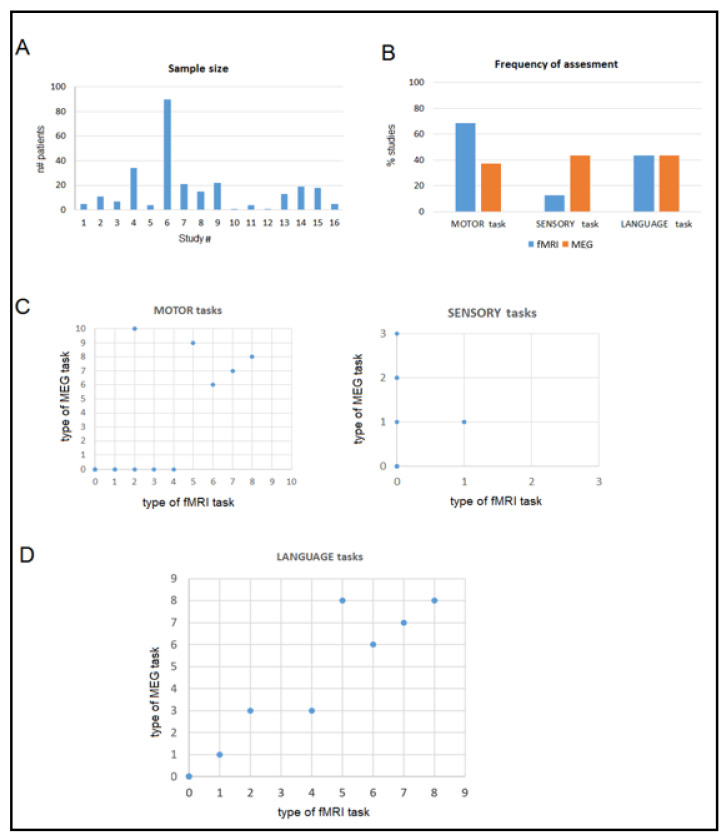
Patients and tasks of the selected studies. (**A**) The number of patients involved in each selected study. (**B**) The percentage of studies that investigated the motor, sensory, and language functions by fMRI and MEG. (**C**,**D**) The correspondence between fMRI and MEG concerning the adopted task/stimulation, respectively for the motor and sensory function and for the language function (same number is attributed to same task or group of tasks across the studies).

We identified 16 studies, reporting findings from a highly variable number of patients (in the range of one to 90 patients). Sample sizes were generally small, with a median of 12 subjects (Figure 2A). The study results are reported in Table 1 and Table 2 in chronological order, from 1995 till present, with the purpose of providing a temporal evolution of the use of the two techniques. Indeed, improvements in both devices, processing algorithms and methods have enabled the increased reliability of the results from each approach. As commented on before, the improved reliability for fMRI can be attributed to the increased tomograph magnetic field strength (1.5 T vs. 3 T), the use of different EPI versus the classical gradient-echo sequence, and increased spatial resolution, with a consequent reduction in partial-volume effects. Concerning MEG, the reliability of solutions to the inverse problem have increased with the progressive use of whole-head devices, possessing an increased number of sensors, by combining both magnetometers and gradiometers, and by processing data by using alternative methods to single ECD.

The reliability of the findings from each technique was most frequently derived from the comparison with intra-operative mapping [11,23,28,44,45,46,48,49]. Alternatively, the confirmation of a good pre-operative assessment was given in the case of successful imaging-guided resection [47,50,51,53,54,55] and/or when the findings matched with those achieved by other techniques, such as the Wada test (in the case of laterality assessment; [47,48,50]) or stimulation methods including transcranial magnetic stimulation (TMS; [51]). Two studies had slightly different purposes than pre-operative planning. In one study [52], pre-operative mapping was performed to help plan the appropriate dose of chemotherapy and, in the other [56], to assess post-operative neuroplasticity changes associated with each language in bilinguals.

The second variable was the domain assessed. There was a dissociation between the frequency of motor and sensory tasks: a higher number of fMRI studies, compared to MEG studies, used motor localizers; vice versa, a higher number of MEG studies, compared to fMRI studies, used sensory localizers (Figure 2B). In contrast, the frequency of language assessments was comparable between fMRI and MEG studies. We observed that the earliest studies (up to 2003) focused exclusively on the central sulcus/sensorimotor cortex localization. The first studies investigating language were published in 2006 [47,48]; interestingly, all the selected studies focusing on language explored expressive language functions, although MEG was observed to be inaccurate at assessing these functions [57]. Overall, studies found general agreement between the results achieved by the two approaches independently.

Nevertheless, several cases of discrepancy were reported. Excluding partially different localizations attributable to the use of different tasks for the fMRI and MEG assessments (and, in case, for intra-operative mapping, too; see Figure 2C,D), the authors attributed the discrepancies to several factors. Most frequently, the ability to accurately detect functional activation was lower for fMRI, mainly represented by altered neurovascular coupling due to the close presence of a draining vein or edema associated with the pathological condition [23,28,55]. Pathology represented a notable limitation for fMRI. Inoue et al. [23] observed fMRI inaccuracy for the patients but not for the healthy controls and the possibility of restored accurate localization following surgery. In one case [47], a decreased BOLD contrast was observed in the vicinity of large and/or high-grade lesions.

In another case [49], fMRI failed in motor activation localization in the pre-central gyrus, but localized the source in the post-central gyrus. The authors commented that one potential limitation of this technique resides in its capacity to detect non-primary areas recruited to perform a given task [23], whereas MEG can discriminate between activation in primary and association areas. However, another study [53] observed the same phenomenon for MEG, too. Given that these authors carried out tumor resection based on pre-operative mapping and that the patient did not develop post-operative motor disturbances despite complete pre-central gyrus excision, it is crucial to understand whether apparently erroneous displacement should be interpreted, rather, in terms of functional reallocation of the investigated function in some case.

On the other hand, a few studies reported issues related to MEG acquisition. In detail, MEG could not be performed or provided poor results in cases of hemiparesis [44,45]—although fMRI could still show a residual activation [45]—or was difficult to execute with regularity for the proposed task [47]. In other cases, it produced severe artifacts due to the presence of dental implants [44,54].

Finally, we observed that, in three studies, pre-operative assessment by fMRI–MEG combination was further improved by the integration with relatively recent techniques, such as TMS [51] or diffusion-weighted/diffusion-tensor imaging [46,50,51]. TMS was used to confirm findings and to detect, within the fMRI functional network, areas that were essential to perform language tasks. Diffusion-weighted/diffusion-tensor imaging, on the other hand, was used to identify the integrity and potential dislocation of the subcortical connections in lesions involving the white matter; moreover, they helped in interpreting the functional imaging results. Several studies reported reliable and valuable pre-operative assessment by both TMS [58,59] and diffusion-weighted/diffusion-tensor imaging [60,61], claiming for their additional contribution and recommended use, at least when feasible.

### Quantitative Comparison

A few studies provided a quantitative computation of potential discrepancies between fMRI–MEG localizations. Kober et al. [45] reported comparable sensorimotor area localizations by fMRI and MEG, although the Euclidean distance between them was not considered to be negligible, both for the sensory (15 ± 5 mm) and motor (10 ± 5 mm) activation. In Korvenoja et al. [49], who similarly aimed to localize the central sulcus, the Euclidean distance was inferior, with MEG dipoles located within 1–6 mm from the nearest fMRI activation voxel and 6–36 mm from the fMRI maximal *z*-score voxel. Zimmerman et al. [54] computed the Euclidean distance by adopting distributed inverse methods instead of single ECD and observed that, in 40% of the case of discrepancy, the mean distance was 3.8 mm for sensory fields with sLORETA and 7.4 mm for motor fields with MNE. In a subsequent study [55], the authors focused on language functions and used dSPM; they reported a mean Euclidean distance of 10.58 mm when taking into account the activation maxima and 9.06 mm for centroids. Although the first two studies underlined discrepancies in these findings, the latter concluded that, overall, there was a good correspondence between the pertaining results.

A thorough evaluation of accuracy based on the comparison with intra-operative mapping was carried out by Ellis and coworkers [11]. The authors computed the Youden’s J statistic [62], which combines sensitivity and specificity to estimate the overall accuracy of each approach. The statistic was computed at several distances between the fMRI/MEG activation and the DCS point. In detail, the authors found the highest J statistic for both motor and language fMRI at a 5 mm distance (J = 0.21 and 0.28, sensitivity = 0.34 and 0.31, specificity = 0.87 and 0.97, respectively) and for MEG at a 40 mm distance for motor mapping (J = 0.28, sensitivity = 0.86, specificity = 0.42) and a 15 mm distance for language mapping (J = 0.28, sensitivity = 0.56, specificity = 0.71). At the standard 10 mm distance used in the literature, there was a poor overlap between the localizations detected by the two approaches. However, their combination increased the accuracy for both the motor (J = 0.29, sensitivity = 0.62, specificity = 0.67) and language (J = 0.40, sensitivity = 0.69, specificity = 0.71) localizations. Overall, for motor mapping, the maximum J statistic (i.e., 0.35, sensitivity of 0.55, specificity of 0.79) was achieved using a 5 mm distance threshold for fMRI and a 15 mm distance threshold for MEG. For language mapping, this was achieved at the above-reported 10 mm threshold.

First, these findings suggest that when interpreting the results in reference to direct stimulation points, the distance threshold has to be taken into account. Depending on this distance and on the monitored function (motor or linguistic), either fMRI or MEG can be more accurate overall and that is necessary to find a compromise between sensitivity and specificity. Second, in light of these considerations, a combination of the two techniques appears as the best solution to assure reliable findings.

## 4. Conclusions

The gold standard in brain tumors surgery is represented by the awake procedure, during which an intra-operative mapping of the function of interest is performed. Nevertheless, for those patients for whom this procedure is not feasible or refused by the patients themselves, it is fundamental to achieve the most accurate function localization in order to prevent permanent post-operative sequelae. Adopting recent devices and processing procedures and algorithms is essential to increase the pre-operative mapping reliability. Additional cautions should be used. For instance, in order to reliably detect residual activity, especially in the case of fMRI, it is useful to adopt individual thresholds (i.e., by adjusting the *p* value when needed) when analyzing the data. However, this could be still insufficient to ensure a reliable pre-operative assessment [63]. In general, relying on a single technique might be risky in the case of surgery under general anesthesia, although, in combination with the assessment of the actual functional status of the patient, it could provide a general guideline for surgery planning.

In fact, in light of these limitations but also the mapping potential of both techniques, the majority of the included studies, both earlier [45,46,47,48] and more recent [11,51,52,53,54,55,56], underscored their complementarity: using both of them could enable a complete and reliable view of the brain activity. For instance, Kamada et al. [48] proposed the complementary use of both techniques to investigate language functions. In fact, MEG versus fMRI seemed to better detect temporoparietal activity (associated more with receptive language), whereas in several studies it seemed to less accurately detect signals from the frontal areas (associated more with expressive language, see [57]).

In the introductory part, we commented on how the availability of MEG devices is very limited worldwide, due to their cost and maintenance burden. However, more practical and economical MEG devices have been recently developed, which could increase MEG feasibility. For instance, optically pumped magnetometer (OPM) devices seem promising, although recording is still constrained to a limited signal bandwidth [20]. Importantly, they do not require expensive cryogenic components for maintenance. This system has reached a sensitivity comparable to that of SQUID sensors, but with increased signal power given that the sensors can be directly placed over the scalp. Alternatively, fMRI should be combined with other techniques; for instance, pre-operative assessment by fMRI could be confirmed by using non-invasive stimulation with more feasible TMS [51,58,59]. Further technological improvement will facilitate, in the near future, the distributed access to user-friendly devices which will enable even more accurate patient-tailored pre-operative surgical planning.

## Figures and Tables

**Table 1:** Selected studies.

Authors	Sample Size	Lesion	Aim	Reference	Additional Relevant Examinations	Results	fMRI–MEG Agreement
Morioka et al. (1995) [28]	5 (4 F)	Various (glioma, AVM, cyst, metastasis, MG) in peri-rolandic areas	Pre-operative sensorimotor cortex localization	Intra-operative (median-nerve) somatosensory stimulation (phase-reversal algorithm)	MR angiography, motor-evoked potentials	fMRI failed correct localization in 4/5 patients due to edema or large cortical vein activation; MEG localized the sensory cortex correctly in all patients (agreement with intra-operative mapping in 4/5 patients)	N/A
							
Inoue et al. (1999) [23]	11 (7 F)	Various (MG, lymphoma, cavernoma) in frontal/parietal areas	Pre-operative CS localization	Intra-operative (median-nerve) somatosensory stimulation (phase reversal and maximum amplitude algorithms) in 10 pts	MR angiography	By fMRI, the CS could not be defined in 7/11 affected hemispheres (wider activation areas including large veins). MEG defined it correctly in all, with agreement with intra-operative mapping	Coincidence in 9/11 patients for the affected hemisphere and in all for the healthy patients (and in all controls). In case of discrepancy (n = 2), intra-operative mapping confirmed MEG- and not fMRI-defined sulcus to be correct (in one case, fMRI defined the sulcus correctly following tumor resection)
							
Nimsky et al. (1999) [44]	7 (6 F)	Various (glioma, cyst, metastasis, MG) adjacent to the motor cortex	Pre-operative central region localization	Intra-operative (median-nerve) somatosensory stimulation (phase-reversal algorithm)	None	Correct CS identification by both in all patients confirmed by intra-operative mapping (but MEG could not be performed in 2 patients because of severe artifacts or hemiparesis)	Correspondent results in all patients for both motor and sensory activations
							
Kober et al. (2001) [45]	34 (24 F)	Various (glioma, angioma, cavernoma, cyst, epilepsy, MG, metastasis) adjacent to the motor cortex	Pre-operative central region localization	Intra-operative (median-nerve) somatosensory stimulation (phase-reversal algorithm)	None	CS localization successful in all cases using either fMRI or MEG (94% with sensory fMRI, 97% with motor fMRI, 97% with sensory MEG, and 79% with motor MEG). The latter could not be used in patients with hemiparesis, in which a small fMRI activity was detected. No clear superiority of one modality over the other	Overall, correspondent results, although localization significantly differed (Euclidean distance) for both sensory (15 ± 5 mm) and motor (10 ± 5 mm) activations
							
Kamada et al. (2003) [46]	4 (1 F)	Various (glioma, cavernoma) involving the motor area	Pre-operative central region localization	Intra-operative (median-nerve) somatosensory stimulation (phase-reversal algorithm)	Anisotropic diffusion-weighted MRI	fMRI-MEG integration correctly identified the CS	N/A
							
Grummich et al. (2006) [47]	90	Various (glioma, AVM, metastasis, MG, other) in frontal or temporo-parietal areas of the dominant hemisphere	Pre-operative language mapping	Imaging-guided resection without post-operative sequelae; comparison with Wada test for 10 pts	Wada test for 10 pts	Broca and Wernicke’s areas correctly localized in all patients; however, fMRI data were too weak in the case of high-grade/large lesions and MEG data could not be used for tasks not performed regularly by the patients	Overall congruence in 77% of cases with greater agreement for Wernicke’s than Broca’s area
							
Kamada et al. (2006) [48]	1 (M) + 20 as controls	Glioma in the right insula (in frontal/temporal areas for controls)	Pre-operative language mapping	Imaging-guided resection with mild post-operative language deficits + Wada test + control pts	Wada test	Both fMRI and MEG showed left-hemisphere dominance for expressive tasks and right-hemisphere dominance for receptive tasks (in agreement with findings from Wada test)	Concordant findings (not directly compared) for the word categorization task
							
Korvenoja et al. (2006) [49]	15 (9 F)	Various (glioma, cavernous hemangioma, MG) close to the primary sensorimotor cortex	Pre-operative CS localization	Intra-operative (median-nerve) somatosensory stimulation (phase-reversal algorithm) and/or cortical stimulation (phase reversal)	None	MEG correctly localized the CS in all patients (N20m goodness-of-fit = 88.8–98.2%) and fMRI in 73% (in 27% it localized it in the post-central gyrus)	MEG dipoles within 1–6 mm Euclidean distance from nearest fMRI activation voxel and 6–36 mm from the fMRI maximal z-score voxel
							
Kamada et al. (2007) [50]	22 (13 F)	Glioma in frontal/temporal areas of the language-dominant hemisphere	Pre-operative language mapping	Intra-operative cortical and subcortical mapping during execution of language tasks for 2 pts	Wada test, diffusion-tensor imaging	Both identified language areas and hemispheric language dominance correctly	N/A
							
Choudhri et al. (2013) [51]	1 (F)	Glioma in the left inferior peri-rolandic cortex	Pre-operative facial motor area and language mapping	Imaging-guided resection; TMS	TMS, diffusion-tensor imaging and tracking	Both identified bilateral (but predominantly left-sided) language activation; agreement between MEG and TMS in localizing early somatosensory response; overall agreement between fMRI and TMS (slight discrepancy attributed to the use of different tasks)	N/A
							
De Martin et al. (2017) [52]	4 (2 F)	Metastasis in the right-hand motor cortex	Pre-operative motor activity localization and dose planning	N/A	None	Both localized the functional areas, enabling reduced irradiation during CyberKnife treatment; in one pt, MEG could map functional area within the tumor	N/A
							
Izutsu et al. (2017) [53]	1 (M)	Glioma in the right precentral gyrus	Pre-operative primary motor area localization	Imaging-guided resection without post-operative sequelae	None	Both showed motor activation shift to the post-central gyrus (and contralesional pre-central gyrus)	N/A
							
Zimmermann et al. (2019) [54]	13 (4 F)	Glioma or AVM/hemangioma close to the sensorimotor cortex	Pre-operative sensorimotor cortex localization	Imaging-guided resection with general deficit improvement + 3 (1 F) healthy controls	None	fMRI–MEG agreement in the detection of functional reorganization in five pts. In one pt, MEG data were affected by severe artifacts	60% of MEG localizations mapped within fMRI activations (the remaining localized at a mean Euclidean distance of 3.8 mm for sensory fields with sLORETA and 7.4 mm for motor fields with MNE)
							
Ellis et al. (2020) [11]	19	Various (glioma, cavernoma, metastasis) close to eloquent areas	Pre-operative motor and language mapping and quantitative assessment of its accuracy	Intra-operative somatosensory stimulation (phase-reversal algorithm) and direct cortical stimulation. Computation of the Youden’s J statistics to assess imaging mapping sensitivity and specificity (at a given distance between intra-operative and imaging localizations)	None	Accuracy highly dependent on distance to direct cortical stimulation site: highest accuracy (J statistic) for both motor and language fMRI at a 5-mm distance and for MEG at 40-mm distance for motor mapping and 15 mm distance for language mapping. fMRI–MEG combination increased accuracy.	Poor overlap between locations identified by each modality at the most commonly used 10-mm distance
							
Zimmermann et al. (2020) [55]	18 (8 F)	Various (glioma, cavernoma, MG) in the left, language-dominant hemisphere	Pre-operative language mapping	Imaging-guided resection with general deficit improvement + 3 (1 F) healthy controls	Diffusion-tensor imaging	fMRI could not identify the activity in 5/13 language areas in 3/18 patients (with vascular/hemorrhagic alterations), whereas MEG provided strong activations	Excluding areas in which fMRI could not identify activity, congruent activations (mean Euclidean distance = 10.58 mm for activation maxima and 9.06 mm for centroids)
							
Quiñones et al. (2021) [56]	5 (all M)	Glioma (all low-grade) in brain areas involved in bilingual language	Language mapping changes in bilinguals between pre- and post-surgery	N/A	None	Both revealed post-operative reorganization taking place for the two languages differently	Complementary and even convergent findings between the alpha/theta longitudinal indexes and fMRI longitudinal lateralization indexes

Note. AVM = arterial-vascular malformation; CS = central sulcus; MG = meningioma; MNE = minimum-norm estimate; N/A = non-available information; sLORETA = standardized low-resolution brain electromagnetic tomography; TMS = transcranial magnetic stimulation.

**Table 2:** Methodological details of fMRI and MEG acquisition and processing information in the selected studies.

	fMRI						MEG			
Authors	Tomograph	Imaging Sequence	Spatial Resolution	Task	Design	Activation Map Definition	Equipment	Task	Source Localization	Co-Registration to MRI
Morioka et al. (1995) [28]	1.5 T	EPI	FOV = 240 × 180 mm^2^, matrix = 256 × 128, slice thickness = 7 mm	Self-paced hand clenching	N/A	Identification of hyperintense activated region	37-channel neuro-magnetometer	Finger (no. 1, 2, and 5) stimulation (ISI = 400–500 ms, sampling rate = 520 Hz, filtering: 1–55 Hz)	ECD (N20 current peak)	ECD superimposition on MR images
										
Inoue et al. (1999) [23]	1.5 T	Gradient-echo EPI	FOV = 240 × 240 mm^2^, matrix = 64 × 64, slice thickness = 10 mm	Hand clenching once/sec (30 sec/block)	Three activation and three resting blocks (10 EPI volumes each)	Cross-correlation (coefficient > 0.06 or >0.08)	MR-linked 122-channel system	Median nerve stimulation (200 stimuli, sampling rate = 1280 Hz, filtering = 0.03–400 Hz)	ECD (N20m current peak)	ECD superimposition on MR images
										
Nimsky et al. (1999) [44]	1.5 T	EPI	FOV = 200 × 200 mm^2^, matrix = 64 × 64 (interpolated 128 × 128), slice thickness = 3 mm	Motor activation: hand clenching (once/sec); sensory activation: finger tactile stimulation (ISI = 800 ms)	Three activation and three resting blocks (10 EPI volumes each)	Cross-correlation (coefficient > 0.65 for motor and >0.55 for sensory activation)	2 × 37-channel bio-magnetometer	Motor activation: brisk index finger flection (100 stimuli, ISI = 3000–5000 ms, sampling rate = 520.8 Hz, filtering = 1–100 Hz); sensory activation: see fMRI (200 stimuli, sampling rate = 1041.7 Hz, filtering = 1–200 Hz)	Single ECD (first peak for motor activation and M30 for sensory activation; correlation > 0.95)	ECD superimposition on MR images (by contour-fit algorithm)
										
Kober et al. (2001) [45]	1.5 T	EPI	FOV = 200 × 200 mm^2^, matrix = 64 × 64 (interpolated 128 × 128), slice thickness = 3 mm	Motor activation: hand clenching (once/sec); sensory activation: finger tactile stimulation (ISI = 800 ms)	Three activation and three resting blocks (10 EPI volumes each)	Cross-correlation (coefficient > 0.65 for motor and >0.55 for sensory activation)	2 × 37-channel bio-magnetometer	Motor activation: brisk finger flection (100 stimuli, ISI = 3000–5000 ms, sampling rate = 520.8 Hz, filtering = 0.1–100 Hz); sensory activation: see fMRI (200 stimuli, ISI = 800 ms, sampling rate = 1041.7 Hz, filtering = 1–200 Hz)	Single ECD (P35m current peak; correlation > 0.95 or + current localization by spatial filtering if <0.95)	ECD superimposition on MR images (by contour-fit algorithm)
										
Kamada et al. (2003) [46]	1.5 T	EPI	FOV = 300 × 300 mm^2^, matrix = 128 × 128, slice thickness = 5 mm (2.5 mm gap)	Self-paced finger tapping (approximately once/sec)	Three activation and four resting blocks (five EPI volumes each)	Cross-correlation (*Z* > 3.5)	204-channel bio-magnetometer	Median nerve stimulation (200 stimuli, ISI = 211 ms, filtering (averaged signals) = 1–70 Hz)	Single ECD (N20m current peak; correlation > 0.95 and confidence volume < 200 mm^3^)	ECD superimposition on MR images
										
Grummich et al. (2006) [47]	1.5 T	EPI	Voxel size = 3 mm^3^	Visually presented tasks selected according to the patient’s clinical features: word-reading task (300 stimuli), sentence-reading task (61 stimuli), picture-naming task (75 stimuli), verb-generation task, arithmetic task. ISI = 900–2000 ms	Six activation blocks interleaved with resting blocks	Cross-correlation (coefficient > 0.03, *p* < 0.000045, *k* ≥ 6)	2 × 37-channel bio-magnetometer	See fMRI (but with ISI = 1200–2300 ms, sampling rate = 520.8 Hz, filtering = 0.1–200 Hz + 0.03–1 Hz)	Single ECD (correlation > 0.94 or current-density reconstruction for lower correlations)	ECD superimposition on MR images (by contour-fit algorithm)
										
Kamada et al. (2006) [48]	1.5 T	EPI	FOV = 260 × 260 mm^2^, matrix = 64 × 128, slice thickness = 4 mm (2 mm gap)	Two tasks: expressive task (verb generation from acoustically presented nouns (ISI = 1600–2400 ms)) and receptive task (word categorization of visually displayed words (ISI = 1800–2200 ms)).	Three activation and four resting blocks (five EPI volumes each)	*Z*-score estimation (clusters with *Z* > 2.2 and *k* > 10)	204-channel bio-magnetometer	Word categorization of visually displayed words (150 stimuli, ISI = 2800–3200 ms, filtering (averaged signals) = 0.01–30 Hz)	Single ECD (+multiple-current estimates to confirm results; correlation > 0.90)	ECD superimposition on MR images
										
Korvenoja et al. (2006) [49]	1.5 T	Gradient-echo EPI	FOV = 256 × 256 mm^2^, matrix = 128 × 128, slice thickness = 3 mm	Self-paced hand clenching	Alternated activation and resting blocks (91–128 EPI volumes)	*Z*-score estimation (clusters with *Z* > 1.6 and neighborhood weighting = 0.427)	122- or 306-channel magnetometer	Median-nerve stimulation (about 200 stimuli, sampling rate = 987 Hz, filtering = 0.03–320 Hz)	ECD (N20m current peak)	ECD superimposition on MR images
										
Kamada et al. (2007) [50]	1.5 T	EPI	FOV = 260 × 260 mm^2^, matrix = 64 × 128, slice thickness = 4 mm (2 mm gap)	Verb generation from acoustically presented nouns (ISI = 1600–2400 ms)	Three activation and four resting blocks (five EPI volumes each)	*Z*-score estimation (clusters with Z > 2.2)	204-channel bio-magnetometer	Word categorization of visually displayed words (150 stimuli, ISI = 2800–3200 ms, filtering (averaged signals) = 0.01–30 Hz)	Single ECD (N400m current peak; correlation = > 0.85)	ECD superimposition on MR images
										
Choudhri et al. (2013) [51]	3 T	N/A	N/A	Motor tasks: tongue movement and lip puckering; language tasks: silent word generation, object naming, and sentence completion	Alternated activation and resting blocks (five volumes each)	N/A	248-magnetomer system	Motor activation: index finger stimulation; language activation: auditory word recognition (sampling rate = 508 Hz, frequency range = 0.1–20 Hz)	N/A	Dipole superimposition on MR images
										
De Martin et al. (2017) [52]	3 T	Gradient-echo EPI	Voxel size = 2.5 mm^3^	Two tasks: hand lifting and hand lowering (21 sec/block)	20 activation blocks alternated with resting blocks	GLM (voxels with *p* < 0.01 and Bonferroni correction)	306-channel neuro-magnetometer	Brisk hand extension following a visual stimulation (at least 100 stimuli, sampling rate = 1000 Hz, filtering = 0.01–100 Hz)	dSPM (movement-related field peak; threshold = 80% of maximum value)	ECD superimposition on MR images (by iterative closest-point algorithm)
										
Izutsu et al. (2017) [53]	3 T	Single-shot EPI	Voxel size = 3 mm^3^ (3.75 mm gap)	Hand grasping (15 sec/block)	Three alternated activation and resting blocks	GLM (clusters with *t* > 4.0)	160-channel system	Hand grasping following either visual or auditory stimulation (ISI = 5500 ms, sampling rate = 1000 Hz, filtering > 200 Hz)	Event-related desynchronization (synthetic aperture magnetometry—a beamforming approach—to compute power changes in theta, alpha, beta, and low- and high-gamma bands)	N/A
										
Zimmermann et al. (2019) [54]	1.5 T	EPI	FOV = 192 × 192 mm^2^, matrix = 64 × 64, slice thickness = 3 mm	Two tasks: flection-extension of all digits or toes	Three activation and three resting blocks (30 EPI volumes each)	Linear correlation (coefficient > 0.03, *p* < 0.000045, and clusters with *k* ≥ 4)	248-magnetomer system	Flection-extension of digits two to five or toes (two separate tasks) following a tactile stimulation (300 stimuli, ISI = 3600 ms, sampling rate = 678 Hz, filtering = 0.01–200 Hz)	MNE, dSPM, sLORETA	ECD superimposition on MR images
										
Ellis et al. (2020) [11]	3 T	Single-shot fast-field EPI	Voxel size = 3 mm^3^	Motor tasks: finger tapping, foot movement, and lip pursing (following an acoustic signal (ISI = 1000 ms)); language task: word reading (15 sec/block)	10 activation and 10 resting blocks (five EPI volumes each)	Thresholded by eye	306-channel system (102 magnetometers and 204 planar radiometers)	Motor tasks (11 patients): finger tapping, foot movement, and face movement, following an auditory stimulation (about 120 stimuli, ISI = 3500–4000 ms); language task (11 patients): word reading (ISI = 3000 ms) (for both: sampling rate = 1000 Hz, filtering = 0.01–330 Hz)	Single ECD (Nelder-Mead nonlinear search algorithm; ≥90% variance and 95% confidence volumes < 3 cm)	ECD superimposition on MR images (by iterative closest-point algorithm and using a spherically symmetric single-conductor head model)
										
Zimmermann et al. (2020) [55]	1.5 T	EPI	FOV = 192 × 192 mm^2^, matrix = 64 × 64, slice thickness = 3 mm	Two tasks: verb conjugation and sentence building (up to 150 stimuli each)	Three activation and three resting blocks (30 EPI volumes each)	Linear correlation (coefficient > 0.03, *p* < 0.000045, and clusters with *k* ≥ 4—or until signal appearance)	248-magnetomer system	Two tasks: verb-conjugation task (300 stimuli, ISI = 3000 ms) and sentence-building task (300 stimuli, ISI = 2000 ms) from visually presented words (for both: sampling rate = 678 Hz, filtering = 0.1–200 Hz (0.03–95 Hz for averaged signals) + 50- and 60-Hz Notch)	dSPM	ECD superimposition on MR images
										
Quiñones et al. (2021) [56]	3 T	EPI	voxel size = 2 mm^3^	Naming task (object- and action-naming) in the two known languages (44 stimuli for each category, ISI = 2000–8000 ms)	event-related design (368 EPI)	Robust weighted least-squares regression with FDR correction (*p* < 0.05, height Threshold: *p* < 0.001, and *k* > 50)	360-channel system	See fMRI (but ISI = 2000–3000 ms, sampling rate = 1000 Hz, filtering = 0.01–330 Hz)	Time-frequency representations (cluster-based permutation approach to compute power changes in theta, alpha, and beta bands)	ECD superimposition on MR images

Note. dSPM = dynamical statistical parametric mapping; ECD = equivalent current dipole; EPI = echo-planar imaging; FDR = false discovery rate; GLM = general linear model; *k* = number of voxels; ISI = inter-stimulus interval; MNE = minimum-norm estimate; N/A = non-available information; sLORETA = standardized low-resolution brain electromagnetic tomography.

## Data Availability

The authors accessed PubMed, Web of Science, and Scholar databases up to the end of 2022.
